# Annotations

**Published:** 1890-10-11

**Authors:** 


### Annotations.

**Costers to the Rescue.**

Doncaster has a perpetual round-about, on which its youthful members love to disport themselves. All the year round the steam horses work under the management of Mr. Tuby, but one day in the year the steam horses work for the infirmary and dispensary. On that day the volunteer band comes down to the ground and adds to the attraction, and the wooden horses with wide open nostrils whirl round and round at extra speed. The result is £17 sent to the infirmary. But somehow or other this institution has found its way so deeply into the people's hearts that none care to be left out in offering aid for its support; and so Mr. Teal, the proprietor of a hot peas stall, on the same occasion, gave the evening's takings of £1 3s. to the infirmary, and Mr. Richard's ice-cream stand yielded 17s. 3d., and Mr. Cook's galvanic battery 16s. Oh for the pen of Dickens to bring out the pathetic and comic points of this story! Perhaps it was not a very cultivated gathering at the Market Place at Doncaster which rode a round-about and ate peas-pudding and ice-cream; but the thankful hearts and willing hands were there, and the people who used the infirmary, with true British pride, did their best to support it. It is a story which reflects enormous credit on the people of Doncaster, and also on the infirmary; for if that institution had not endeared itself to its patients, you may be sure no wooden horses would have swung round in its cause.

**Patients' Dinners.**

At a time when the food supply of nurses has been the subject of general controversy something may be said on the patients' behalf. In some hospitals the serving of the dinner and other meals leaves much to be desired. It is the custom to send up the joints from the kitchen into the ward, where they are carved at a table in sight of the patients, and are then distributed by the nurses. Another plan is to have all the dinners carved in the kitchen, and to have them sent up in portions or messes, the latter being generally the most appropriate description of the unappetising appearance they often present. We happened to be at the Swansea Infirmary recently during the dinner-hour. Each patient was brought the most temptingly served food we have ever seen offered to hospital patients. It appears that the plan is to send up the food from the general kitchen to the ward kitchen, where the plates are kept hot until the patients are ready for their dinner, when the head nurse apportions it to each patient, and is so enabled to meet to a great extent the tastes, and to humour the appetites of those in her charge. The Swansea Hospital maintains 130 beds, the average daily number occupied last year being 80. Of course this is a relatively small number, but there is no reason at all why with a little management every hospital patient, no matter what the size of a particular institution may be, should not have his dinner served as appetisingly and as nicely as at Swansea. We congratulate the committee upon the efficiency of the house surgeon, Mr. Hopkins, an old University College man, and the matron, Miss Bellars, who both show the keenest interest in everything which concerns the efficiency and well-being of the hospital.

**Harvest Festivals and Hospitals.**

It was our privilege last week to be present at a Harvest Festival in a hospital chapel. On the 2nd inst. the chapel of the Gloucester Infirmary was most tastefully decorated by the nurses, not only with flowers, but with corn and fruit, so that the general effect was excellent. We have always felt that it is a contradiction in terms for the decorations of a church at a harvest festival to consist of flowers only, the fruits of the earth being conspicuous by their absence. The chapel at Gloucester was crowded with patients and nurses, the *locum tenens* house surgeon, Mr. Collins, with the matron, Miss Yeats, being also present. The chaplain, whose name we do not know, is evidently a faithful steward, and his ministrations have had a marked effect for good upon all connected with the hospital. The sermon was preached by the deputy-chairman of the Gloucester Infirmary, who is also the chairman of the Board of Guardians. It is customary to have a collection at the harvest festivals, and this year it was given to the Society for the Propagation of the Gospel in Foreign Parts. As the preacher said, it is desirable at a thanksgiving festival that the alms of the congregation should be given to some object outside the particular hospital with which the chapel may be connected. We would suggest for the consideration of the chaplains of all the hospitals throughout the country, the propriety of having a collection each year at the harvest festivals in aid of the Donation Bonus Fund of the National Pension Fund for Nurses. If this idea was generally followed it would result in some hundreds of pounds being given annually for this useful purpose, and would enable the council of the fund to provide for many worthy women who from no fault of their own fall out of the nursing ranks and become disabled for work at an early age. We may add that we rejoice to see what a charming dining-room the Gloucester Infirmary Committee have supplied for their nurses, the sitting-room being also good, and that some thoughtful person has provided an excellent library from which the nurses can select books to read during their hours of leisure.

**The Winter Session.**

The entries at the various medical schools throughout the country will probably show a larger entry than any previous year. As we went to press last week representatives of the various schools were delivering the introductory addresses. What medical man does not remember the enthusiasm with which he entered upon his career; how anxious he was to secure a particular part, and that a good one; how pleasant were the greetings of his fellows amongst the first year's men! Of late years the school authorities have shown an active desire to promote the comfort and provide for the accommodation of the medical students. The medical colleges are becoming the rule rather than the exception, and they provide board, lodging, and companionship at a fair but reasonable rate. Some men will always prefer the independence of private lodgings to the relative comforts of the residential college. Still, so much liberty is permissible that few members of the rising generation will probably care to follow the example, in this respect, of the ardent spirits of rougher times. In any case, the school authorities are to be congratulated upon the many and great improvements they have everywhere introduced. Parents may now send their sons to the hospital with an assured feeling that they will be properly looked after and cared for. It is true that one correspondent has called our attention to a personage whom he defines as a jew clothier and money-lender who haunts the exterior of several of the large hospitals with the object of inducing the medical students to accept a little loan. Our inquiries have led us to believe that the worthy in question is more intent upon selling clothes than anything else. Whether he does manage to distribute a little cash on the excellent security which most medical students can so readily afford seems doubtful, but if he does we can only hope that he will try to collect the money through one of the courts of this country when no doubt he will get his deserts. Athletics have made great progress amongst students where they were always much in vogue. Many contend that no athlete can read sufficiently. He is always training or exercising and so is never quiet except when he requires rest and sleep. He excites a great interest among his fellow students, and many of them flock to the football and other contests on Saturday afternoons. They become so excited about athletics in consequence that they discuss the events of Saturday until Tuesday or Wednesday, give more or less attention to their work on Wednesday or Thursday, and then begin to discuss and eagerly anticipate the contests of the forthcoming Saturday. These contentions, when made seriously, read as, they no doubt to a great extent are in reality, as sheer nonsense. We are convinced that the man who takes care to have sufficient exercise is, as a rule, the one of all others who gets on best with his work in the end. Of course, the enthusiasm of youth is proverbial and discretion comes, though not always, with increasing years. Still the medical student transplanted from the country to town ought to be encouraged to engage in athletics, and indeed in anything which will take him away from the hospital and out of the City to fresh air and exercise. The medical student has not had as much attention in these columns as he deserves. We hope, however, to rectify this omission in the near future.

